# Cytoplasmic Expression of the ALS/FTD-Related Protein TDP-43 Decreases Global Translation Both *in vitro* and *in vivo*

**DOI:** 10.3389/fncel.2020.594561

**Published:** 2020-12-08

**Authors:** Santiago E. Charif, Luciana Luchelli, Antonella Vila, Matías Blaustein, Lionel M. Igaz

**Affiliations:** ^1^Instituto de Fisiología y Biofísica “Bernardo Houssay”, (IFIBIO-Houssay), Grupo de Neurociencia de Sistemas, Universidad de Buenos Aires y Consejo Nacional de Investigaciones Científicas y Técnicas (CONICET), Buenos Aires, Argentina; ^2^Instituto de Biociencias, Biotecnología y Biología Traslacional, Departamento de Fisiología, Biología Molecular y Celular, Facultad de Ciencias Exactas y Naturales, Universidad de Buenos Aires, Buenos Aires, Argentina

**Keywords:** TDP-43, frontotemporal dementia (FTD), amyotrophic lateral sclerosis (ALS), transgenic mice, protein synthesis, animal model, proteinopathy

## Abstract

TDP-43 is a major component of cytoplasmic inclusions observed in neurodegenerative diseases like frontotemporal dementia (FTD) and amyotrophic lateral sclerosis (ALS). To further understand the role of TDP-43 in mRNA/protein metabolism and proteostasis, we used a combined approach with cellular and animal models overexpressing a cytoplasmic form of human TDP-43 (TDP-43-ΔNLS), recapitulating ALS/FTD features. We applied in HEK293 cells a method for labeling *de novo* translation, surface sensing of translation (SUnSET), based on puromycin (PURO) incorporation. While control cells displayed robust puromycilation, TDP-43-ΔNLS transfected cells exhibited reduced ongoing protein synthesis. Next, by using a transgenic mouse overexpressing cytoplasmic TDP-43 in the forebrain (TDP-43-ΔNLS mice) we assessed whether cytoplasmic TDP-43 regulates global translation *in vivo*. Polysome profiling of brain cortices from transgenic mice showed a shift toward non-polysomal fractions as compared to wild-type littermates, indicating a decrease in global translation. Lastly, cellular level translational assessment by SUNSET was performed in TDP-43-ΔNLS mice brain slices. Control mice slices incubated with PURO exhibited robust cytoplasmic PURO signal in layer 5 neurons from motor cortex, and normal nuclear TDP-43 staining. Neurons in TDP-43-ΔNLS mice slices incubated with PURO exhibited high cytoplasmic expression of TDP-43 and reduced puromycilation respect to control mice. These *in vitro* and *in vivo* results indicate that cytoplasmic TDP-43 decreases global translation and potentially cause functional/cytotoxic effects as observed in ALS/FTD. Our study provide *in vivo* evidence (by two independent and complementary methods) for a role of mislocalized TDP-43 in the regulation of global mRNA translation, with implications for TDP-43 proteinopathies.

## Introduction

TAR DNA-binding protein 43 (TDP-43) is a ubiquitously expressed, predominantly nuclear protein with multiple roles in RNA processing (Buratti et al., 2001; Gao et al., 2018; Nussbacher et al., 2019). In neurodegenerative diseases such as amyotrophic lateral sclerosis (ALS) and frontotemporal dementia (FTD), a key finding is that pathological TDP-43 accumulates in the cytoplasm, forming aggregates (Arai et al., 2006; Neumann et al., 2006; Ito et al., 2017). Nevertheless, how this mislocalization contributes to pathogenesis is still poorly understood.

Although the most extensively described function of TDP-43 has been its role in mRNA splicing, there has been reports consistent with a role for this protein in translational regulation. A proteomic study in HEK293 cells showed that TDP-43 interact extensively with proteins of the translational machinery (Freibaum et al., 2010) and *in vitro* downregulation of TDP-43 alters global translational yield through a SKAR-dependent mechanism (Fiesel et al., 2012). Moreover, the ribosomal protein RACK1 seems to be a key mediator of the interaction of TDP-43 with the translational machinery (Russo et al., 2017).

Using inducible transgenic mice, we demonstrated that expression of a cytoplasmic form of TDP-43 (termed TDP-43-ΔNLS) recapitulates multiple features of TDP-43 proteinopathies of the ALS/FTD spectrum, including progressive neurodegeneration, gliosis, global gene expression changes and behavioral abnormalities encompassing the motor, cognitive and social domains (Igaz et al., 2011; Alfieri et al., 2014). Other animal models with cytoplasmic TDP-43 expression, covering from *Drosophila* to mammalian systems, also support the idea that this mislocalization event might be pathogenically relevant (Miguel et al., 2011; Walker et al., 2015; Wang et al., 2015; Yin et al., 2019).

While most studies have focused on the regulation of global or specific translation using cell lines or primary neurons in culture, the consequences of *in vivo* TDP-43 manipulation (specifically, of its cytoplasmic form) are virtually unknown. In this work, we investigated both *in vitro* and *in vivo* if cytoplasmic expression of TDP-43 modifies global protein synthesis, using two different approaches, polysome profiling and surface sensing of translation (SUnSET) technique.

## Materials and Methods

### Surface Sensing of Translation (SUnSET) in Cultured Cells

#### Cell Culture and Transfection

Myc epitope-tagged hTDP-43-ΔNLS construct was generated in pcDNA 5/To plasmid (Invitrogen) using restriction sites BamHI and XbaI, as previously described (Winton et al., 2008). Human embryonic kidney HEK293T cells were grown in high glucose (4.5 g/L glucose) Dulbecco's modified Eagle's medium (DMEM, Gibco) supplemented with 10% fetal bovine serum, penicillin/streptomycin (100 units/ml and 100 μg/ml respectively, Thermo Fisher Scientific), and 110 mg/L of sodium pyruvate (Thermo Fisher Scientific) in a 37°C humidified incubator containing 5% CO_2_. Cells (5 × 10^5^ per well) were plated on poly-D-lysine-coated 12 mm glass coverslips in a 6-well plate, and grown for 24 h before transfection. Lipofectamine 2000 reagent (Invitrogen) was used for transfection of constructs (empty vector as control or hTDP-43-ΔNLS), according to the manufacturer's instructions. After 48 h, medium was replaced with fresh medium with or without puromycin (10 μg/ml, P7255, Sigma) for 20 min and washed with PBS.

#### Immunocytochemistry

Cells on coverslips were fixed in 4% paraformaldehyde in phosphate-buffered saline (PBS) for 10 min, permeabilized with 0.2% Triton X-100 (Sigma) in PBS for 10 min, blocked with 5% powdered milk in PBS for 2 h, and incubated overnight with primary antibody at 4°C. Primary antibodies used in this study (diluted in 5% powdered milk in PBS) were as follows: anti-total-TDP-43 [1:5000, rabbit polyclonal anti-TDP-43 1038C (Igaz et al., 2008)] and anti-puromycin (1:1000 dilution, MABE343, clone 12D10, Millipore). Primary antibodies were visualized with secondary antibodies conjugated with Alexa Fluor 488 and Alexa Fluor 594 (1:500, donkey anti-mouse and donkey anti-rabbit IgG, respectively, Jackson Immunoresearch), and nuclei were detected using Hoescht 33258 (Sigma). Coverslips were mounted in gelatin-coated slides using Fluoro-gel (Thermo Fisher Scientific) and stored at 4°C. Images were obtained with a Zeiss AxioImager 2 microscope equipped with APOTOME 0.2 structured illumination, using a Hamamatsu Orca Flash 4.0 camera. Four areas per image were analyzed using ImageJ software (version 1.52a). Mean puromycin and TDP-43 signals were calculated and normalized to empty vector (+PURO) group signal.

### Animals

hTDP-43-ΔNLS line was generated as described in Igaz et al. (2011). Monogenic tetO-TDP-ΔNLS mice were bred to Camk2a-tTA mice (Jackson Laboratory) generating non-transgenic, tTA monogenic, single tetO-TDP-43 transgenic mice, and bigenic mice expressing hTDP-43-ΔNLS. Breeding mice and pups were treated with 0.2 mg/ml doxycycline hyclate (DOX; sc-204734A; Santa Cruz Biotechnology) in drinking water, to avoid pre-natal and post-natal developmental effects. Transgene expression was activated at weaning (post-natal day 28) by removing DOX from water and mice were analyzed after ~1 month (at post-natal day 60). Tail DNA was used for genotyping, employing primers described in Igaz et al. (2011). The experimental protocol for this study was approved by the National Animal Care and Use Committee of the University of Buenos Aires (CICUAL). All animal procedures were performed according the NIH Guide for the Care and Use of Experimental Animals.

### Polysome Profiling

#### Sucrose Gradient Preparation

Cortices of control (non-transgenic) or hTDP-43-ΔNLS animals were homogenized in cold lysis buffer [15 mM Tris-HCl, pH 8.0, 300 mM NaCl, 15 mM MgCl_2_, 1% Triton X-100, 1 mg/ml heparin, protease inhibitors (Roche) and 0.1 mg/ml cycloheximide (Sigma-Aldrich Inc.)] at a ratio of 500 μl buffer /10 mg tissue. Homogenates were then placed on ice for 10 min and centrifuged at 5,000 rpm for 10 min at 4°C. Supernatant, consisting in cytosolic extract containing polysomes, was collected and protein concentration was determined by bicinchoninic acid (BCA) method (Pierce). Sucrose gradient, ranging from 15 to 50%, were prepared in gradient buffer (15 mM Tris-HCl, pH 8.0, 300 mM NaCl, 5 mM MgCl_2_, 1 mg/ml heparin and 0.1 mg/ml cycloheximide). Briefly, 2.4 ml 50% sucrose solution was added to a tube and chilled for 30 min at −80°C. The procedure was repeated with layers of sucrose solution at lower concentration (41.25, 32.5, 23.75, and 15%) and stored at 4°C if used the next day. 0.5 to 1 mg of cytosolic protein extract were added to each gradient, and centrifuged in a SW40 rotor at 35,000 rpm for 2–4 h, at 4°C. Five hundred microliters aliquots were separated and absorbance at 260 nm was measured (Eppendorf Biophotometer Plus, Germany). A peak area ratio between polysomal (P) and non-polysomal (NP, comprising RNPs and monosomes) fractions was determined for each group.

#### Western Blot

Five hundred microliters of sample from each fraction was subjected to methanol/chloroform protein extraction method and resuspended in sample buffer. Equal amounts of samples were resolved on 10% sodium dodecyl Sulfate polyacrilamyde gel electrophoresis (SDS-PAGE) and transferred onto PVDF membranes (Immobilon-P; Millipore). After transfer, membranes were stained with Ponceau S for total protein load assessment, then blocked in 5% powdered milk in 0.1% Tween-TBS, and incubated in anti-total TDP-43 primary antibody overnight (1038C, 1:20000). Detection of primary antibody was performed with horseradish peroxidase-conjugated secondary antibody (Jackson Immunoresearch), and ECL was used for blot development. Digital images were acquired using a G-BOX Imager (Syngene). Ponceau S staining was used as a control for equal protein loading in our immunoblot analysis of polysomal fractions, since typical housekeeping genes are barely detectable in polysome fractions (Reschke et al., 2013).

### Surface Sensing of Translation (SUnSET) in Brain Slices

#### Tissue Processing

SUnSET labeling in brain slices was adapted from Cook et al. (2014), with some modifications. Brain coronal slices were prepared from 8-week old (1 month off-DOX) hTDP-43-ΔNLS or control mice littermates. Slices were cohort and age matched. Mice were anesthetized with isoflurane and decapitated; brain was removed and chilled ice-cold in 95 O_2_-5% CO_2_-saturated, sucrose-based artificial cerebrospinal fluid (ACSF, 220 mM sucrose, 2.5 mM KCl, 1 mM CaCl_2_, 6 mM MgCl_2_, 1.25 mM NaH_2_PO_4_, 26 mM NaHCO_3_, and 10 mM dextrose). The brain was next glued onto a vibratome stage and acute sagittal slices (400 μm) were cut. Then, the slices were recovered at room temperature for an hour in ACSF (125 mM NaCl, 2.5 mM KCl, 2 mM CaCl_2_, 1.1 mM MgCl_2_, 1.25 mM NaH_2_PO_4_, 26 mM NaHCO_3_, and 10 mM glucose, pH 7.4). After recovery, slices were placed on a fine grid mesh in a petri dish and incubated in ACSF (C- group) or ACSF supplemented with 5 μg/μl puromycin (P7255, Sigma-Aldrich Inc.; C+ or ΔNLS+ groups) at a final volume of 30 ml. Incubation time was 45 min under continuous aeration with carbogen (95% O_2_/ 5% CO_2_). Slices were then rinsed in ACSF and fixated in 4% paraformaldehyde (Sigma-Aldrich Inc.) in 0.1 M PBS for 24 h at 4°C. Next day, slices were washed three times in 0.01 M PBS for 10 min each under continuous agitation in an orbital shaker, incubated with 10% sucrose in 0.01 M PBS for 2 h at 4°C and stored in 30% sucrose-0.01 M PBS at −20°C until use.

#### Immunofluorescence

Brain slices containing the motor cortex [approximately bregma 1.18 mm, (Paxinos and Franklin, 2008)] were selected and washed three times in 0.01 M PBS for 10 min. Permeabilization step was performed with 0.01 M PBS-1% Triton X-100 for 1 h at room temperature. Next, slices were incubated with blocking solution containing 5% normal goat serum in 0.01 M PBS-0.3% Triton X-100 for 1 h at room temperature. Sections were immunolabeled with rabbit polyclonal anti-TDP-43 (1038C, 1:15000) and mouse monoclonal anti-puromycin (MABE343 clone 12D10, Millipore, 1:4000) as primary antibodies; incubation was performed first at room temperature for 3 h followed by 48 h at 4°C. Staining against the neuronal marker NeuN (mouse monoclonal MAB377, Millipore, 1:1000) or human TDP-43 protein (mouse monoclonal 60019-2, Proteintech, 1:10000) was performed in 50 μm slices as described in Alfieri et al. (2014). Then, slices were washed three times for 10 min each with 0.01 M PBS-0.04% Triton X-100 and incubated with Alexa Fluor 594 goat anti-rabbit IgG and Alexa Fluor 488 goat anti-mouse IgG (each at 1:500 dilution, Jackson laboratories) for 4 h at room temperature in darkness. Slices were washed three times for 10 min with 0.01 M PBS-0.04% Triton X-100 and subjected to Hoescht staining (1:2000 dilution in 0.01M PBS) for 30 min at room temperature in darkness. Finally, sections were washed in 0.01 M PBS two times (10 min each) and mounted in gelatin-coated glass slides with 30% glycerol in 0.01 M PBS, covered with glass coverslips, sealed and stored in darkness at 4°C until use. All incubation and wash steps were performed in a 12-well plate under continuous agitation in an orbital shaker.

#### Image Detection and Analysis

Images were obtained with a Zeiss AxioImager 2 microscope equipped with APOTOME.2 structured illumination, using a Hamamatsu Orca Flash 4.0 camera. Two sections of layer 5 cells of motor cortex were analyzed using ImageJ software (version 1.52a). In non-transgenic (control, C) mice incubated with puromycin (C+), each cellular area was defined in the green channel corresponding to puromycin signal, as well as background areas between cells; the same approach was employed in hTDP-43-ΔNLS animals incubated with puromycin (ΔNLS+) but in the red channel (total TDP-43 signal). For non-transgenic mice incubated in ACSF (C-), a circular area centered in each red nucleus and background areas between cells were quantified. Background signal was normalized to individual cell areas, and a ratio between raw cell intensity signal and normalized background signal was calculated. The signal from individual cell populations and averaged signal per animal were compared between experimental groups.

### Statistical Analysis

Data are expressed as means ± SEM. Statistical analysis was performed using a standard unpaired two-tailed student's *t*-test when comparing only two groups on one measure. A one-way ANOVA followed by Tukey's multiple-comparison *post hoc* test was used when comparing three groups. Prism Version 8 (GraphPad Software) was used for statistical analysis and differences were considered to be statistically significant when the probability value was < 0.05.

## Results

**Figure 1:**
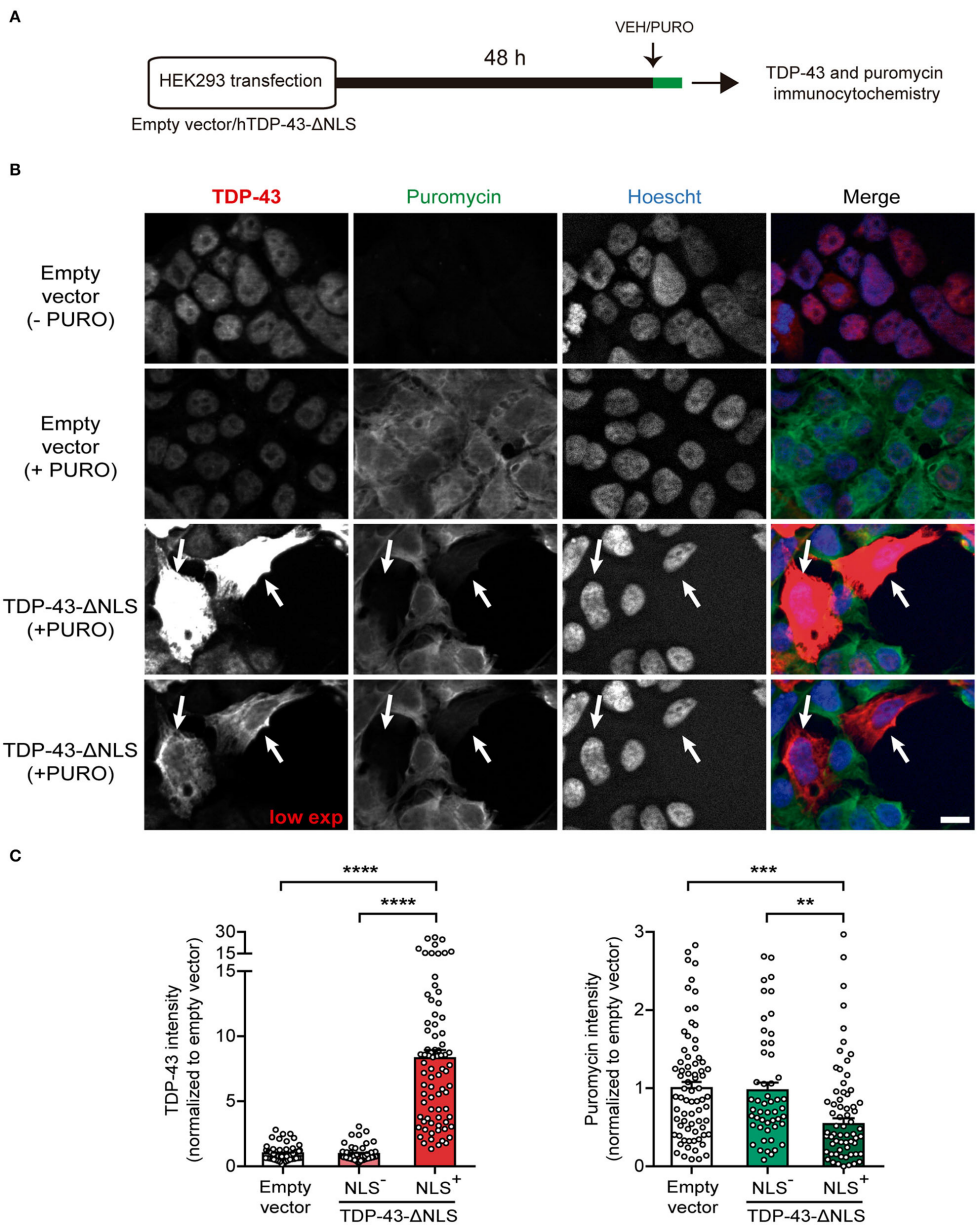
Overexpression of a cytoplasmic form of TDP-43 affects global protein synthesis in HEK293 cells. **(A)** Workflow diagram of single cell puromycilation measurement by SUnSET. HEK293 cells were transfected with empty vector or TDP-43-ΔNLS, and 72 h later, a single 20 min puromycin (+ PURO) or Vehicle (VEH, -PURO) pulse was applied to the cultures. Then, cells were subjected to total TDP-43 and puromycin IF, and Hoescht 33258 was used as a nuclear marker. **(B)** Representative micrographs of total TDP-43 (red) and puromycin (green) IF staining. Control (empty vector) cells treated with vehicle (- PURO) served to assess non-specific staining and no puromycin signal was detected, while control (+ PURO) cells exhibited robust puromycilation. In both control groups, TDP-43 labeling was nuclear (as expected for endogenous expression), while TDP-43-ΔNLS transfected cells showed intense, cytoplasmatic expression. A lower exposure for the TDP-43 channel is also shown. Puromycin staining is visibly decreased in TDP-43-ΔNLS transfected cells (arrows). Scale bar: 10 μm. **(C)** Quantitative analysis of puromycilation signal revealed that cells with cytoplasmic TDP-43 (NLS+) exhibit a decreased ongoing translation. Staining intensity of TDP-43-ΔNLS immunopositive cells show significant differences when compared to puromycin-labeled control cells and non-transfected (NLS-) cells. TDP-43 levels were significantly higher in NLS+ than in the control or non-transfected groups (as expected for transgene overexpression). Data represent mean ± SEM; *n* = 75 cells per condition (from three replicates). One-way ANOVA/Tukey's multiple comparison test: ****P < 0.0001, ***P < 0.001, **P < 0.01.

As a first approach to study at the cellular level the effect of cytoplasmic TDP-43 expression on protein synthesis, we adapted the SUnSET methodology (Schmidt et al., 2009) to measure single-cell puromycilation by immunocytochemistry (Figure 1). Puromycin incorporation into nascent polypeptides enables direct visualization of actively translating ribosomes. We transfected either empty vector or TDP-43-ΔNLS in HEK293 cells and, during the last 20 min of the experiment, applied puromycin (+ PURO) or vehicle (-PURO) to the cultures (Figure 1A). Representative micrographs show immunofluorescence staining using antibodies against total TDP-43 or puromycin (Figure 1B). Control (empty vector) cells treated with vehicle (-PURO) were used to assess non-specific staining and showed virtually no puromycin signal, while control (+PURO) cells displayed robust puromycilation. As expected, TDP-43 staining showed nuclear, endogenous TDP-43 for both control groups, while TDP-43-ΔNLS transfected cells demonstrate abundant, cytoplasmic expression in a proportion of HEK293 cells. Remarkably, analysis of puromycilation signal in TDP-43-ΔNLS transfected cells demonstrates that cells with cytoplasmic TDP-43 (NLS^+^) show a decrease in ongoing protein synthesis (arrows, Figure 1B). Quantification of puromycin staining intensity in TDP-43-ΔNLS positive cells show significant differences against both the puromycin-labeled control cells and TDP-43-ΔNLS negative (non-transfected) cells (Figure 1C). As expected, TDP-43 levels are significantly higher in TDP-43-ΔNLS cells than in the control or non-transfected groups (Figure 1C). Overall, these results demonstrate at cellular resolution that cytoplasmic TDP-43 decreases ongoing protein synthesis in cultured cells.

**Figure 2:**
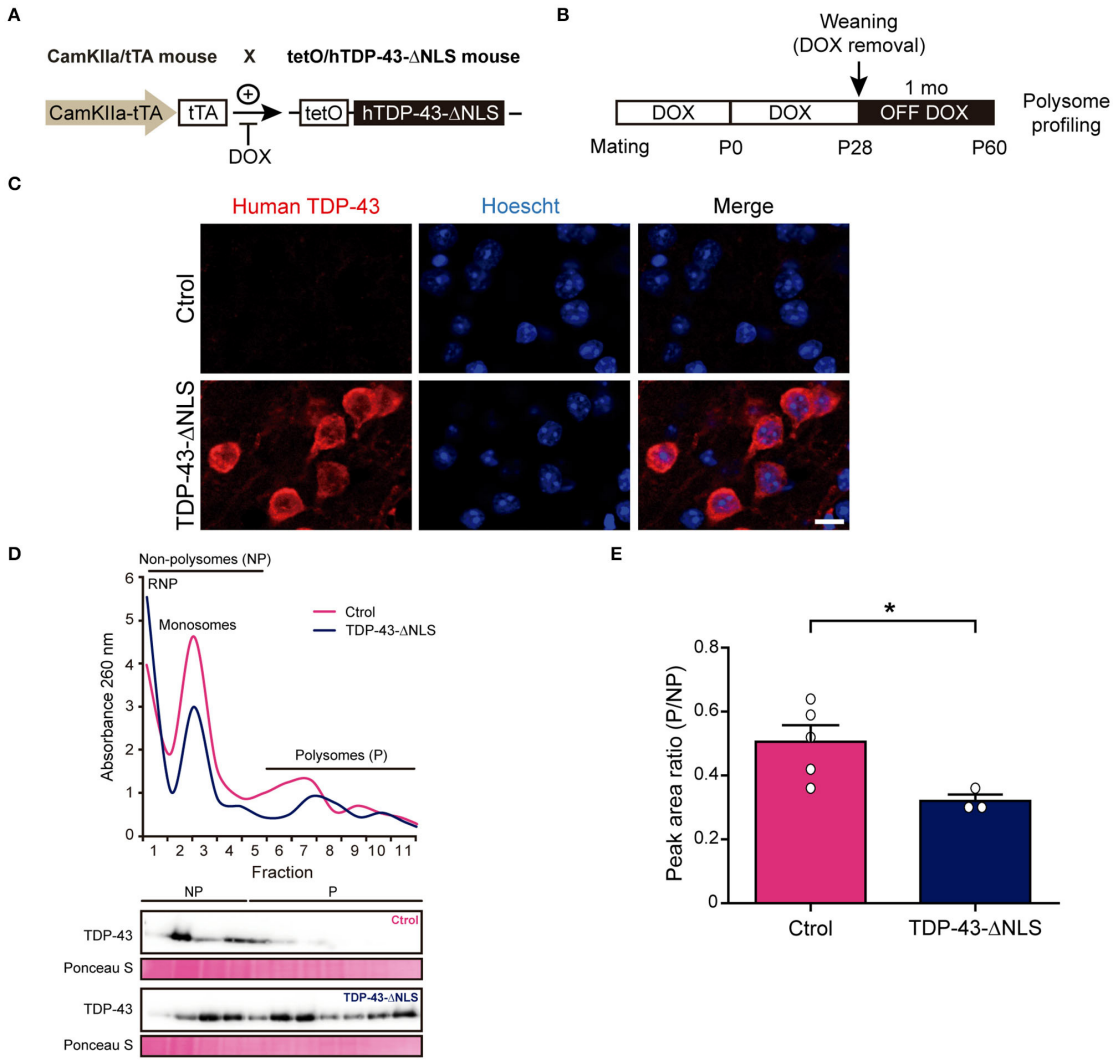
Transgenic TDP-43-ΔNLS mice have an altered polysome profile in brain cortex. **(A)** Generation of hTDP-43-expressing mice. CamKIIa/tTA mice are crossed with tetO/hTDP-43-ΔNLS mice. In bigenic mice, doxicycline (DOX) inhibits tTA binding to the tetracycline–responsive element (tetO), thus repressing hTDP-43 expression. Non-transgenic littermates were used as controls. **(B)** Timeline and experimental workflow. Mating and breeding of animals was carried in the presence of DOX to avoid developmental effects. Transgene expression was induced at weaning (P28) by removing DOX from water (OFF DOX), and mice were analyzed at ~1 month post-induction (P60). Then, brain cortices were subjected to polysome profiling. **(C)** Representative micrographs of human-specific TDP-43 antibody IF in control (Ctrol) and TDP-43-ΔNLS mice at 1 month off DOX. Transgenic animals show robust cytoplasmic expression of the transgene in cortical neurons. Hoescht 33258 was used as a nuclear marker. Scale bar: 10 μm. **(D)** Representative cortical homogenate polysome profiles in non-transgenic (Ctrol) and transgenic (TDP-43-ΔNLS) mice. In TDP-43-ΔNLS animals, the free ribonucleoprotein (RNP) fraction is increased while the translationally active fractions (polysomes, P) are decreased. Immunoblots against total TDP-43 using protein isolated from the individual fractions revealed that overexpression of TDP-43-ΔNLS increases the presence of total TDP-43, especially in the polysomal fractions. Ponceau S was used as a loading control. **(E)** Quantification of the ratio between the areas under the curve for polysomal (P) and non-polysomal (NP) fractions. Transgenic mice display a significant reduction, indicating that global translation in brain cortex is decreased due to cytoplasmic TDP-43 expression. Data represent mean ± SEM; *n* = 5 for Ctrol, *n* = 3 for TDP-43-ΔNLS. *P < 0.05, significantly different from Ctrl mice (Student's *t*-test).

Next, we wanted to extrapolate these results *in vivo*, taking advantage of our inducible human TDP-43-ΔNLS mouse model with CamKIIα-driven transgene expression enriched in forebrain neurons (Igaz et al., 2011). We first aimed to study global protein synthesis by polysome profiling (Figure 2), a method that allows for translational state assessment by differential separation of mRNA-protein complexes in a sucrose gradient (Chasse et al., 2017). TDP-43-ΔNLS or control mice were generated (Figure 2A) and transgene induction was started at P28 to avoid developmental deficits. Animals were sacrificed at P60 (after 1 month of transgene expression), a time point when these mice already develop ALS/FTD-like phenotypes (Igaz et al., 2011; Alfieri et al., 2014; Walker et al., 2015) (Figure 2B). Immunofluorescence analysis of TDP-43-ΔNLS mice demonstrate robust cytoplasmic transgene expression in cortical neurons, as revealed by an antibody that specifically stains human TDP-43 (i.e., the transgene product) but not endogenous mouse TDP-43 (Figure 2C). Polysome profiles from control (non-transgenic) or TDP-43-ΔNLS mice cortical homogenates show an increase in the free ribonucleoprotein (RNP) fraction (low density) and a decrease in translationally active fractions of high density (polysomes) in transgenic mice (Figure 2D). Interestingly, immunoblots using total TDP-43 antibody show that overexpression of TDP-43-ΔNLS leads to an increased presence of TDP-43 in most gradient fractions, but especially in those with higher density. Quantification of the ratio between areas under the curve for polysomal and non-polysomal (i.e., non-translationally active) fractions demonstrate a significant reduction in transgenic mice (Figure 2E), indicating that cortical global protein synthesis is reduced *in vivo* due to cytoplasmic TDP-43 expression.

**Figure 3:**
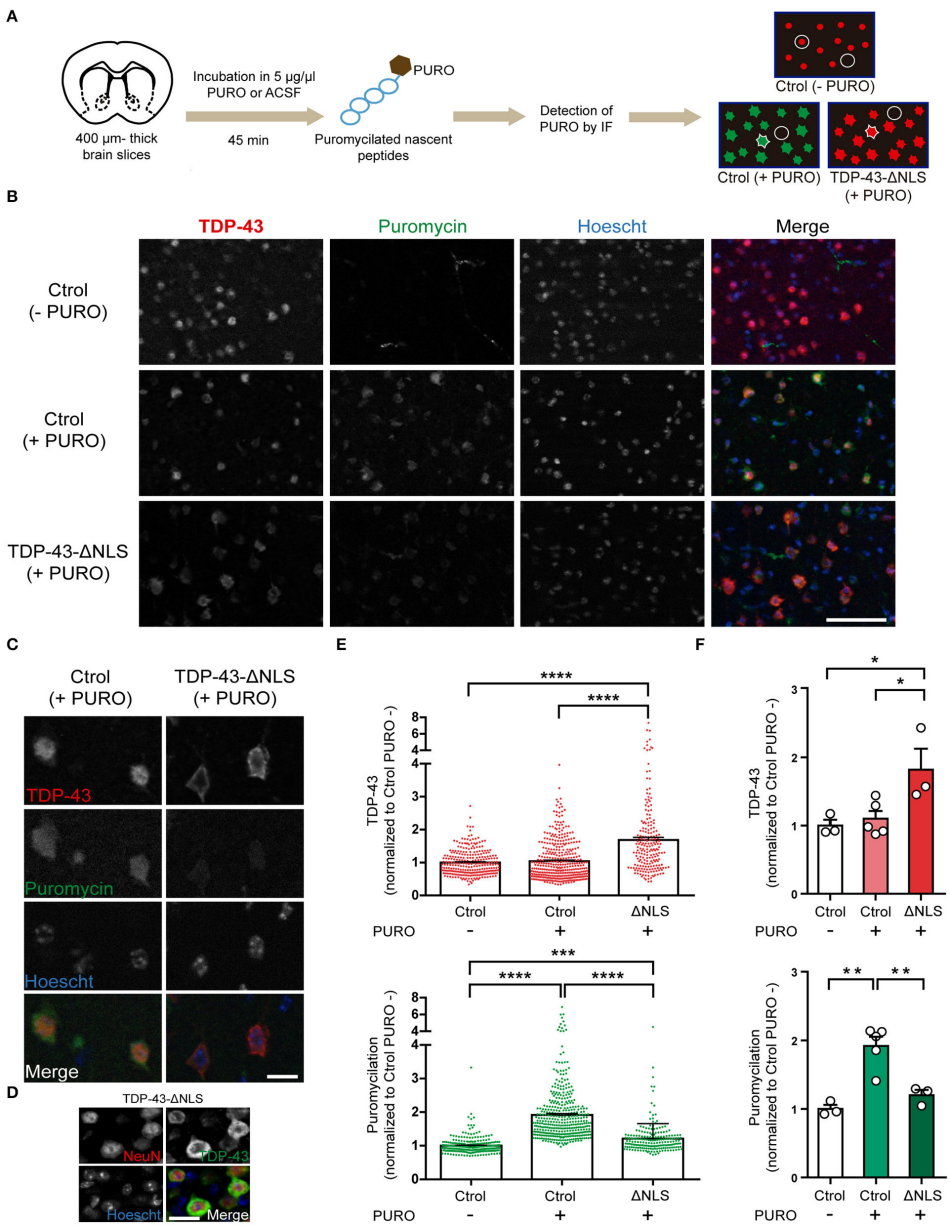
Cortical motor neurons from TDP-43-ΔNLS mice show decreased ongoing protein synthesis *in vivo*. **(A)** Diagram of the puromycilation experiment (SUnSET technique). Coronal brain slices from non-transgenic (Ctrol) and transgenic (TDP-43-ΔNLS) mice were incubated in 5 μg/μl puromycin (+ PURO) or ACSF (- PURO) for 45 min. Detection of nascent puromycilated peptides and total TDP-43 was done by immunofluorescence (IF). Microscopy images of motor cortex (layer 5) were analyzed. In Ctrol (+PURO) mice, cellular areas were defined in the green channel (puromycin signal, cell contour), as well as background areas between cells (circle); for TDP-43-ΔNLS (+PURO) animals the same approach was employed but using the red channel (total TDP-43 signal). For Ctrol (-PURO) mice, an area centered in the red nucleus (circle around the red dot) and background areas (circle) were registered. **(B)** Puromycin (green) and TDP-43 (red) IF in acute cortical slices from Ctrol (-PURO and +PURO) and ΔNLS (+PURO) mice. Hoescht 33258 was used as a nuclear marker. Scale bar: 50 μm. **(C)** Higher magnification images of representative cells as described in **(B)**. Scale bar: 10 μm. **(D)** Representative micrographs of double IF using the neuronal marker NeuN (red) and TDP-43 (green) in cortical sections from TDP-43-ΔNLS mice at 1 month off DOX. NeuN staining show that cells with cytoplasmic TDP-43 staining are neurons. Hoescht 33258 was used as a nuclear marker. Scale bar: 10 μm. **(E)** Fold change (respect to Ctrol -PURO) analysis of TDP-43 (top) and puromycin (bottom) cellular labeling. *n* = 278 for Ctrol (-PURO), *n* = 396 for Ctrol (+PURO), *n* = 203 for ΔNLS (+PURO). **(F)** Average TDP-43 (top) and puromycin (bottom) cell signal for each experimental mice. *n* = 3 for Ctrol (-PURO), *n* = 5 for Ctrol (+PURO), *n* = 3 for ΔNLS (+PURO). One-way ANOVA /Tukey's multiple comparison test; *P < 0.05, **P < 0.01, ***P < 0.001, ****P < 0.0001. Data represent mean ± SEM.

Our polysome profiling data strongly suggest that global translation was decreased in the brains of TDP-43 mice, but this technique does not allow for examination of cell-specific populations. To elucidate if TDP-43-ΔNLS mice display altered ongoing protein synthesis *in vivo* at the neuronal level, we applied the SUnSET method in slices from control or transgenic mice and studied puromycilation levels in neurons from layer 5 of the motor cortex (Figure 3). We evaluated protein synthesis after 1 month of TDP-43-ΔNLS expression, using acute thick slices (400 μm) from coronal vibratome sections. After a short incubation with puromycin, nascent peptides were labeled and detected at the cellular level with anti-PURO immunofluorescence (Figure 3A). Additionally, we used an anti-TDP-43 antibody to identify and evaluate both cells expressing endogenous TDP-43 protein (nuclear), and neurons expressing transgenic TDP-43-ΔNLS (cytoplasmic) for further quantification. Low magnification immunofluorescence images form Layer 5 of the motor cortex show that non-transgenic, control mice (Ctrol) incubated with vehicle (ACSF) instead of puromycin (-PURO) showed normal nuclear staining of endogenous TDP-43 (Figure 3B). As expected, anti-PURO signal was negligible, although some non-specific staining of blood vessels can be observed (this is likely due to remaining IgG in the slices reacting with the anti-PURO Ab raised in mice). Slices from control mice incubated with puromycin display robust cytoplasmic signal in most cells, indicating proper labeling of ongoing protein synthesis. As expected, TDP-43 endogenous levels are also nuclear and with intensity comparable to control (-PURO) group (Figure 3B). TDP-43-ΔNLS mice slices incubated with PURO show the expected pattern of increased cytoplasmic expression of TDP-43, while these cells display a reduction in puromycilation (Figure 3B). A high-magnification view of cortical motor neurons from layer 5 clearly show that cells overexpressing cytoplasmic TDP-43 display reduced levels of anti-PURO signal (Figure 3C). Neuronal identity of cells with cytoplasmic TDP-43 was corroborated by NeuN and TDP-43 double immunofluorescence on thin cortical slices from TDP-43-ΔNLS mice (Figure 3D). Quantification at the cellular level demonstrates that TDP-43-ΔNLS mice display a significant increase in TDP-43 signal intensity compared to both control groups, and that PURO treatment does not affect TDP-43 levels (Figure 3E). Cellular analysis of puromycilation in those same neurons exhibits the expected significant increase in control groups treated with (+ PURO) vs. without (-PURO) puromycin. Importantly, neurons in TDP-43-ΔNLS mice display a drastic reduction in protein synthesis as revealed by anti-PURO signal (Figure 3E). These results are also evident when we pool data from each individual animal, with a significant reduction of puromycin signal in neurons from transgenic mice (Figure 3F).

## Discussion

Protein synthesis is an essential process for all cellular types, but neurons are particularly sensitive to proper control of this metabolic process due to their high polarization and the demands of plasticity mechanisms (Kapur et al., 2017). Several studies have suggested a role for translational dysfunction in a wide range of neurodegenerative diseases, including Alzheimer's, tauopathies, Huntington's, Prion disease, and the ALS/FTD spectrum (Ding et al., 2005; Halliday et al., 2015; Sun et al., 2015; Meier et al., 2016; Russo et al., 2017; Kamelgarn et al., 2018; Zhang et al., 2018; Joag et al., 2019; Koren et al., 2019). In this report, we show both in cultured cells and, more importantly, in cortical neurons *in vivo* that cytoplasmic TDP-43 leads to altered protein synthesis, expanding our understanding of the possible role of this RNA-binding protein (RBP) in ALS/FTD pathogenesis.

There are other examples of RBPs involved in neurological disease (Thelen and Kye, 2019), including FMRP (related to Fragile X syndrome) and FUS (associated with ALS/FTD) (Donlin-Asp et al., 2017; Zhou et al., 2018). FMRP regulates activity-dependent translation of its mRNA cargoes (Davis and Broadie, 2017), and some of its functions are performed in cooperation with other RBPs, including TDP-43 and Staufen 1 (Chu et al., 2019). FUS is similar in several aspects to TDP-43, and recent reports demonstrated that disease-related mutations in FUS can lead to suppression of global (Kamelgarn et al., 2018) or axonal translation (Lopez-Erauskin et al., 2018). However, most of these studies exploring the relationship between neurodegenerative disease-related proteins and translation were performed either in cultured cell lines or in primary cultures derived from different transgenic mouse models. A remarkable *in vivo* study recently showed that TDP-43 and DISC1 co-aggregate in frontotemporal lobar degeneration human samples and animal models, leading to disruption of dendritic local translation and aberrant behavior (Endo et al., 2018).

We show here that TDP-43-ΔNLS expression reduces the amount of puromycin incorporated into translating peptides, and found that although TDP-43-ΔNLS is abundantly localized to polysome fractions, there are fewer polysome-associated mRNAs. This raises the possibility that mislocalized TDP-43 may disrupt mRNA recruitment to polysomes. In addition, the direction of change in translation opposes that seen with TDP-43 knockdown (Fiesel et al., 2012), suggesting that rather than loss of function, there is a gain of toxic function that might be mediated by TDP-43 abnormal expression/localization. Further studies investigating these possibilities are warranted.

What could be the potential players involved in this translational effect mediated by TDP-43-ΔNLS? A report using SH-SY5Y cells has shown that cytoplasmic TDP-43 interacts with both RACK1 and ribosomal protein S6 (rpS6) as demonstrated by immunoprecipitation, and that TDP-43-ΔNLS co-localizes in the cytoplasm with RACK1 and rpS6, suggesting that TDP-43 associates to translational machinery (Russo et al., 2017). RACK1 is an associated ribosomal protein and functions as a docking site for several proteins on the translational machinery; in addition, it has been shown to accumulate in TDP-43 positive inclusions in motor neurons of ALS spinal cord cases (Russo et al., 2017). Other potential candidates are those identified in a proteomic study using HEK293T cells in which a main cluster of TDP-43-interacting proteins include a cytoplasmic “translational” cluster, which contains multiple translation initiation and elongation factors, and ribosomal subunits (Freibaum et al., 2010). Although it is a predominantly nuclear protein, it has been previously shown that TDP-43 shuttles between the nucleus and cytoplasm (Winton et al., 2008), and alteration of cytoplasmic TDP-43 levels might lead to abnormal formation of complexes involved in translation, due to an altered stoichiometric ratio between TDP-43 and its protein and RNA partners. Lastly, TDP-43 has been shown to regulate the alternative splicing of SKAR (Fiesel et al., 2012), a protein that positively affects cap-dependent protein translation (Ma et al., 2008). TDP-43 downregulation leads *in vitro* to a shift in SKAR α/β isoform ratio, which in turn increases global translation (Fiesel et al., 2012). Interestingly, Yang et al. (2014) used a mouse model to show that partial TDP-43 knock-down increased SKAR isoforms with exon 3 excluded, leading to a lower α/β isoform ratio in brain and spinal cord due to altered alternative splicing.

Different mechanisms induced by ALS/FTD-related proteins may lead to translational repression. Pathological forms of these proteins can trigger stress granule formation and/or can activate ER stress-induced pathways (i.e., PERK) leading to translational modulation events such as eIF2α phosphorylation (Cestra et al., 2017). High levels of misfolded proteins stimulate the Unfolded Protein Response (UPR), which tend to stabilize proteostasis and may lead to protein synthesis inhibition. Chronic, sustained activation of these pathways can lead to cell death (Li et al., 2006; Sano and Reed, 2013). ER stress markers have been detected in samples from ALS patients and animal models (Matus et al., 2013); remarkably, ER-stress induced HIPK2 activation and neurodegeneration has been described in TDP-43-ΔNLS mice (Lee et al., 2016). Alleviation of disease-related phenotypes can be achieved in neurodegenerative disease models by pharmacological and genetic interventions which lead to protein translation restoration (Moreno et al., 2013; Halliday et al., 2017). In this line, targeting UPR signaling is an attractive goal, given the variety of proof-of-concept drugs that act at different steps, such as PERK activation (i.e., GSK2606414) or eIF2α signaling (ISRIB), potentially enabling translational recovery (Costa-Mattioli et al., 2007; Kim et al., 2014; Halliday et al., 2015; Radford et al., 2015).

Given that our TDP-43-ΔNLS mouse model recapitulates several FTD/ALS-related behavioral phenotypes (Alfieri et al., 2014), our present results showing global translational inhibition suggest that this alteration can be part of a pathogenic mechanism affecting not only neuronal survival but also multiple behavioral domains. An important step in future studies will be to apply strategies that tend to restore protein synthesis levels and evaluate if the neurodegenerative and behavioral phenotypes of TDP-43 animal models can be ameliorated.

In conclusion, we have shown here by two different approaches that expression of cytoplasmic TDP-43 leads to a decrease in global translation. Although the role of TDP-43 in protein synthesis regulation has been discussed and explored recently, our *in vivo* results underscore its importance as a mechanistic link between the possible pathogenic molecular changes and the clinical and pathological manifestations of TDP-43 proteinopathies.

## Data Availability Statement

The datasets generated for this study are available on request to the corresponding author.

## Ethics Statement

The animal study was reviewed and approved by National Animal Care and Use Committee of the University of Buenos Aires (CICUAL).

## Author Contributions

SC, LL, and LI designed the study. SC and AV performed the cell culture experiments. LL performed the polysome profiling and immunoblot experiments. SC performed *in vitro* and *in vivo* SUnSET studies, image processing, and data analysis. MB provided key reagents, funding, and helped design and analyze the *in vitro* experiments. SC and LI wrote the manuscript. LI conceived and supervised all aspects of the project and obtained study funding. All authors read and approved the final manuscript.

## Conflict of Interest

The authors declare that the research was conducted in the absence of any commercial or financial relationships that could be construed as a potential conflict of interest.

## Abbreviations

ACSF: artificial cerebrospinal fluid
ALS: amyotrophic lateral sclerosis
Ctrol: control
DOX: doxycycline
FTD: frontotemporal dementia
IF: immunofluorescence
mRNA: messenger ribonucleic acid
NP: non-polysomal fraction
P: polysomal fraction
PBS: phosphate buffered saline
PURO: puromycin
RBP: RNA binding protein
RNA: ribonucleic acid
RNP: ribonucleoprotein
SUnSET: surface sensing of translation
TDP-43: TAR DNA-binding protein 43.
